# Nonfunctional parathyroid cyst: case report

**DOI:** 10.1590/S1516-31802009000600012

**Published:** 2010-05-21

**Authors:** Carlos Eduardo Molinari Nardi, Ricardo Adriano Nasser Barbosa da Silva, Cynthia Maria Massarico Serafim, Rogério Aparecido Dedivitis

**Affiliations:** I MD. Resident in General Surgery, Hospital Ana Costa, Santos, São Paulo, Brazil.; II MD. Resident in Head and Neck Surgery, Hospital Ana Costa, Santos, São Paulo, Brazil.; III Undergraduate medical student, Fundação Lusíada - Centro Universitário Lusíada (Unilus), Santos, São Paulo, Brazil.; IV MD, PhD. Full professor, Fundação Lusíada - Centro Universitário Lusíada (Unilus), Santos, São Paulo, and professor, Postgraduate Course on Health Sciences, Hospital Heliópolis (Hosphel), São Paulo, Brazil.

**Keywords:** Parathyroid diseases, Cysts, Parathyroid glands, Calcium, Parathyroid hormone, Doenças das paratireóides, Cistos, Glândulas paratireóides, Cálcio, Hormônio paratireóideo

## Abstract

**CONTEXT:**

Parathyroid cysts are rare clinical and pathological entities, with less than 300 cases reported. The inferior parathyroid glands are most commonly involved, with left-side predominance. Parathyroid cysts may be functional or nonfunctional, depending on their association with hypercalcemia.

**CASE REPORT:**

A 25-year-old man presented a palpable asymptomatic left-side neck mass. Ultrasound revealed a cystic structure contiguous with the left thyroid lobe. Serum ionic calcium was normal. The patient underwent left thyroid lobectomy plus isthmectomy with excision of the cyst. The histological findings revealed a parathyroid cyst. Parathyroid cysts typically present as asymptomatic neck masses, and surgical excision appears to be the treatment of choice.

## RESUMO

**CONTEXTO:**

Cistos de paratireóide são entidades clínicas e patológicas raras, com menos de 300 casos relatados. As glândulas paratireóides inferiores são mais comumente envolvidas, com predomínio do lado esquerdo. Cistos de paratireóide podem ser funcionais ou não, dependendo de sua associação com hipercalcemia.

**RELATO DE CASO:**

Um homem de 25 anos apresentou-se com massa cervical esquerda palpável assintomática. A ultrassonografia revelou uma estrutura cística contígua com o lobo tireoidiano esquerdo. O cálcio iônico sérico estava normal. O paciente foi submetido a lobectomia esquerda com istmectomia e excisão do cisto. Os achados histopatológicos revelaram cisto de paratireóide. Cistos de paratireóide tipicamente se apresentam como massas cervicais assintomáticas e a ressecção cirúrgica parece ser o tratamento de escolha.

## INTRODUCTION

Parathyroid cysts are rare clinical and pathological entities, with less than 300 cases reported in the world literature. It has been reported that they occur in 0.5% of parathyroid disease cases and represent 1% of all cystic lesions of the neck.^1^ In 1880, Sandstrom reported the first parathyroid cyst.^2^ These cysts commonly occur in the fourth and fifth decades of life,^3^ with a female to male ratio of 2.5:1.^1^ The inferior parathyroid glands are most commonly involved, with left-sided predominance. Parathyroid cysts may be functional or nonfunctional, depending on their association with hypercalcemia. The clinical manifestation may consist of a solitary thyroid nodule or a neck mass.^3^ The treatment options include ultrasound-guided aspiration. However, carcinoma arising in the parathyroid cysts has been reported. Thus, surgical removal of all cysts should be strongly considered.^4^ The purpose of this study was to describe a case of a patient with a parathyroid cyst.

## CASE REPORT

The patient was a 25-year-old man with a six-month history of asymptomatic left-side neck mass. At the physical examination, two painless masses of sizes 2.5 x 1.0 mm and 1.0 x 1.0 mm were palpable. Ultrasound revealed a 28 x 19 x 8 mm cystic structure contiguous with the left thyroid lobe and a thyroid nodule. Fine-needle aspiration biopsy was performed, resulting in findings of squamous epithelial cells that suggested a branchial cyst. Thyroid nodule biopsy was suggestive of hyperplasia or adenoma. Thyroid function tests, including triiodothyronine, thyroxin and thyroid-stimulating hormone were normal.

**Figure 1.**
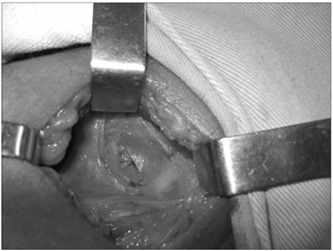
Surgical field showing the large cyst.

**Figure 2.**
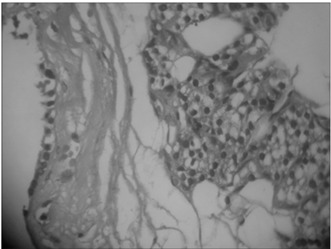
Typical parathyroid tissue seen in the cyst wound. Hematoxylin and eosin, 400 X.

One year later, ultrasonography was performed again, and it revealed cystic growth, now measuring 35 x 26 x 16 mm. Surgery was indicated. The serum ionic calcium concentration was 1.25 mg/dl (1.16-1.32 mmol/l), parathyroid hormone was 35 pg/ml (11-67 pg/ml), thyroid-stimulating hormone was 1.21 mIU/ml (0.4-4.0 mIU/ml) and free T4 was 0.95 ng/dl (0.8-1.9 ng/ml). The patient underwent neck exploration and left thyroid lobectomy plus isthmectomy, with excision of the parathyroid cyst (Figure 1). The histological findings revealed a parathyroid cyst (Figure 2). The parathyroid hormone level after surgery was 11.5 pg/ml, while the ionic calcium was 1.17 mg/dl.

## DISCUSSION

Parathyroid cysts typically present as asymptomatic neck masses,^2^ as in the patient in the current study, although cases of compromised airways and recurrent laryngeal nerve palsy secondary to large cysts have been reported.^2^ Table 1 shows the published papers relating to parathyroid cysts, according to the databases. The most common presentation is a cystic lesion during a neck incision to treat a thyroid nodule.^1^ Parathyroid cysts may be functional or nonfunctional depending on their association with hypercalcemia.^3^

At physical examination, parathyroid cysts tend to be soft, mobile, nontender masses, usually located in the lower part of the neck but can arise at any site between the jaw and the mediastinum.^5^ The diagnostic workup should include a thorough head and neck examination, fine-needle aspiration, ultrasound and thyroid scan. Ultrasound may reveal a nonspecific cystic structure.^3^ On radioiodine thyroid scans, parathyroid cysts appear as areas of absent uptake.^1^ Computed axial tomography scan and magnetic resonance imaging may be indicated when there is a solitary neck mass that is not diagnosed by other methods.^3^ Percutaneous needle aspiration of parathyroid cysts reveals crystal-clear fluid, which is highly suggestive of the diagnosis. Elevated parathyroid hormone levels in the cystic fluid confirm the diagnosis,^2^ but do not indicate that the cyst is functioning^1^. The differential diagnosis should include thyroglossal duct cyst, branchial cleft cyst, thyroid adenoma, and parathyroid carcinoma.

The treatment for parathyroid cyst includes aspiration, injection of sclerosing agents and surgical excision.^3^ Fine-needle aspiration under ultrasound guidance with cystic fluid assay for parathyroid hormone levels represents the approach of choice for both diagnosis and the initial treatment, since this is safe, easy and repeatable.^1^ This method is more effective on nonfunctioning cysts.^3^ Intracystic tetracycline injection may also be used on patients with recurrence; however, this has been associated with neck pain, neurotoxicity and recurrent nerve palsy due to leakage of the sclerosing agents through the disrupted thin cyst wall.^1^ Surgical excision appears to be the treatment of choice for functioning parathyroid cysts^3^ and in cases of repeated recurrence.^1^ Complications caused by removal of parathyroid cysts include hypocalcemia, hemorrhage, hypercalcemic crises, tetany and recurrent laryngeal nerve palsy.^3^

**Table 1.** Published papers relating to parathyroid cysts, according to database

Database	Search strategy	Results	
PubMed	“Parathyroid Diseases” as key word AND “Cyst” as word	165	12 original articles 1 historical article 3 reviews 143 case reports 3 letters to the editor 3 comments on previous articles
Lilacs	“Parathyroid Diseases” as key word	4	1 original article 3 case reports
Cochrane Library	“Parathyroid” AND “Cyst” as words	0	0
